# Efficacy and safety of gemcitabine plus docetaxel in Japanese patients with unresectable or recurrent bone and soft tissue sarcoma: Results from a single-institutional analysis

**DOI:** 10.1371/journal.pone.0176972

**Published:** 2017-05-10

**Authors:** Masanobu Takahashi, Keigo Komine, Hiroo Imai, Yoshinari Okada, Ken Saijo, Masahiro Takahashi, Hidekazu Shirota, Hisatsugu Ohori, Shin Takahashi, Natsuko Chiba, Takahiro Mori, Hideki Shimodaira, Chikashi Ishioka

**Affiliations:** 1 Department of Medical Oncology, Tohoku University Hospital, Aoba-ku, Sendai, Miyagi, Japan; 2 Department of Clinical Oncology, Institute of Development, Aging and Cancer, Tohoku University, Aoba-ku, Sendai, Miyagi, Japan; 3 Department of Cancer Biology, Institute of Development, Aging and Cancer, Tohoku University, Aoba-ku, Sendai, Miyagi, Japan; University of South Alabama Mitchell Cancer Institute, UNITED STATES

## Abstract

**Background:**

Combination therapy with gemcitabine and docetaxel has been reported to be a good therapeutic strategy for patients with soft tissue sarcoma. The aim of the present study was to analyze the efficacy and toxicity of gemcitabine with docetaxel in Japanese patients with advanced bone and soft tissue sarcoma.

**Patients and methods:**

We retrospectively analyzed the effect of gemcitabine and docetaxel therapy on overall response, progression-free survival, overall survival, and toxicity in 42 patients with bone or soft tissue sarcoma who had received the therapy between October 2006 and September 2015, at Tohoku University Hospital.

**Results:**

The median age was 55 years; 23 patients were men, and 19 were women. Eight had bone sarcoma and 34 had soft tissue sarcoma. Forty patients (95%) had previously been treated with one or more chemotherapeutic regimens. The overall response rate was 6.9% and the disease control rate was 55%. The median progression-free survival was 2.3 months and the median overall survival was 14.3 months. Grade 3 or more neutropenia and febrile neutropenia were observed in 74% and 4.8% of all patients, respectively.

**Conclusion:**

The response rate was lower and myelosuppression was more frequently observed than in other previous reports. On the other hand, most of toxicities were enough manageable. In addition, some patients had long survival with a good response. Our study supports the notion that gemcitabine and docetaxel therapy is a good therapeutic option for treating patients with advanced soft tissue sarcoma as well as bone sarcoma, also in Asian populations.

## Introduction

Sarcoma is a rare tumor of mesenchymal cell origin, accounting for about 1% of all adult malignancies. It is estimated that annually, about 3,300 and 12,000 patients are newly diagnosed with bone and soft tissue sarcoma, and about 1,500 and 5,000 patients die from these diseases, respectively, in the United States of America [1]. In particular, soft tissue sarcoma is a heterogeneous group of tumors, composed of more than 50 histological subtypes such as undifferentiated pleomorphic sarcoma, liposarcoma, leiomyosarcoma, synovial sarcoma, malignant peripheral nerve sheath tumors, and many other rarer cancers [2]. Soft-tissue sarcoma appears everywhere in the body including the head and neck, extremities, internal organs, and retroperitoneum. In general, when the disease is localized to a primary region, surgical resection is the best therapeutic option for curing the disease. In some cases, radiotherapy is performed with or without chemotherapy before or after surgery to increase the curative resection rate and/or to decrease the recurrence rate, depending on tumor size, the extent of tumor invasiveness, the existence of a wide margin, or histological grade or subtypes.

In unresectable or recurrent soft-tissue sarcoma, chemotherapy is the main treatment option. Single cytotoxic agents such as doxorubicin [3], ifosfamide [4], dacarbazine [5], epirubicin [6], gemcitabine [7], and temozolomide [8], or the combination regimens of doxorubicin and ifosfamide [3], doxorubicin and dacarbazine [9], ifosfamide and epirubicin [4], and gemcitabine and vinorelbine [10] or docetaxel [11, 12], have been widely used for patients with soft tissue sarcoma. Among these drugs, although there have been a few large phase III studies, doxorubicin has remained as the most frequently used first line drug treatment, with overall response rates (RR) of 12–24% [2, 13, 14].

For second-line therapy, new drug choices have recently emerged. Pazopanib, a multi-targeting tyrosine kinase inhibitor against vascular endothelial growth factor receptors, platelet-derived growth factor receptors, and KIT, was shown to significantly prolong the progression-free survival (PFS) of patients with soft tissue sarcoma as compared with placebo (median PFS, 4.6 vs. 1.6 months; hazard ratio, HR, 0.31; P < 0.0001) in the phase III study PALETTE [15]. Trabectedin has recently been reported to be effective in patients with translocation-related sarcoma who were previously treated with standard regimens (median PFS, 5.6 vs. 0.9 months with best supportive care; HR, 0.07; P < 0.0001) in a randomized phase II trial [16]. Another more recent phase III trial has shown that for metastatic liposarcoma or leiomyosarcoma trabectedin is superior to dacarbazine for extending PFS (median 4.2 vs. 1.5 months; HR, 0.55; P < 0.001) but not for extending overall survival (OS, median, 12.4 vs. 12.9 months; HR, 0.87; P = 0.37) in second-line therapy or later [17]. Furthermore, in a recent phase III study, eribulin was shown to prolong OS as compared with dacarbazine (median OS, 13.5 vs. 11.5 months; HR 0.77; P = 0.017), but not PFS (median PFS, 2.6 vs. 2.6 months; HR 0.88; P = 0.23), for the third-line therapy or later of patients with advanced liposarcoma or leiomyosarcoma [5].

There are increasing new drug choices for soft-tissue sarcoma, but except for doxorubicin, these drugs have shown only limited efficacy, particularly in terms of tumor shrinkage, mostly with a less than 10% RR. Among the current drug regimens, gemcitabine and docetaxel combination (GD) therapy has a relatively high RR in salvage line therapy. A US randomized phase II trial analyzing patients with various soft tissue sarcomas revealed that GD exhibited a RR of 16% compared with 8% for gemcitabine alone [12]. More specifically, GD had an even higher RR of 53% in patients with leiomyosarcoma of the uterus or other organs, as compared with other subtypes of sarcoma, in another US phase II study [11]. In a French retrospective analysis, RR was 24% in uterine leiomyosarcoma, but 10% in other leiomyosarcomas [18]. Similarly, in a more recent French randomized phase II study, the RR was higher for leiomyosarcoma of uterus than for leiomyosarcoma of other organs (24% vs. 5%) [19]. A small Japanese phase II study reported that a response was observed in three of eight (38%) patients with uterine leiomyosarcoma [20]. Despite the lack of phase III studies, these phase II studies suggest that GD is a promising regimen in soft-tissue sarcoma, possibly particularly in uterine leiomyosarcoma, at least as second- or later-line therapy. Phase III studies to compare GD and current standard doxorubicin therapy are required for establishing this regimen for unresectable or recurrent sarcoma. In addition, the efficacy and safety of GD in soft tissue sarcomas other than uterine leiomyosarcoma have not been well established in Asian populations.

In bone sarcomas such as osteosarcoma and Ewing sarcoma, GD therapy is one of the options for second-line therapy for patients with metastatic disease, although there is even less evidence for this than for soft tissue sarcoma. For instance, a retrospective analysis by Navid et al. showed that in 22 children and young adults with bone or soft tissue sarcoma including osteosarcoma, Ewing sarcoma, malignant fibrous histiocytoma, chondrosarcoma, and undifferentiated sarcoma, RR was 29% and the toxicity was manageable [21]. These results, together with similar results from other recent phase II and retrospective studies [22–24], may support the usefulness of GD in bone sarcoma as well as soft tissue sarcoma. However, since these studies included only small numbers of patients and there is a lack of phase III studies, accumulation of more clinical data, ideally from prospective phase III studies, is warranted to establish the usefulness of GD in bone sarcoma as well.

The aim of this study was to reveal efficacy and safety of GD therapy for Japanese patients with advanced bone or soft tissue sarcoma, through a retrospective and single-institutional analysis.

## Patients and methods

### Patients

A total of 42 patients with unresectable or recurrent bone or soft tissue sarcoma were included in this study, who had received GD therapy in Department of Medical Oncology, Tohoku University Hospital, between October 2006 and September 2015. Patients with measurable and/or non-measurable lesions and those with non-measurable lesions alone were included in this study. Clinical outcomes as described below were retrospectively analyzed through the use of medical records.

### Treatment

Patients received GD therapy composed of 900 mg/m^2^ of gemcitabine by intravenous infusion for 30 min on days 1 and 8, and 70 mg/m^2^ of docetaxel by intravenous infusion for 90 min on day 8, every 3 weeks. The dose of docetaxel was decided as 70 mg/m^2^, because the maximum approved dose of docetaxel was 70―75 mg/m^2^ in Japan when this study started. The doses of gemcitabine and docetaxel were reduced to appropriate levels (approximately a 20% reduction of both gemcitabine and docetaxel) when grade 4 hematological toxicities, grade 3 or 4 non-hematological toxicities, or other toxicities that were considered to affect continuation of the therapy, were observed. Granulocyte-colony stimulating factor (G-CSF) was not administered for prophylactic use. G-CSF was therapeutically used when grade 4 neutropenia or grade 3 febrile neutropenia was observed. The therapy was continued until disease progression (PD), severe toxicities leading to discontinuation of therapy, or patients’ desire to suspend therapy.

### Evaluation of tumor response rate, progression-free survival, overall survival, and toxicity

Tumor response was evaluated by computed tomography every 2 to 3 months, according to the Response Evaluation Criteria in Solid Tumors version 1.1 [25]. Overall RR was defined as the number of patients with a complete response (CR) or a partial response (PR), divided by the number of all patients with measurable lesions. The disease control rate (DCR) was defined as the number of patients with CR, PR, or stable disease (SD), divided by the number of response-evaluable patients.

PFS was defined as the period from the initiation of GD therapy to PD. OS was defined as the period from the initiation of gemcitabine and docetaxel therapy to death from any cause. The final update for survival was performed on April 1, 2016.

Toxicities were evaluated according to the Common Terminology Criteria for Adverse Events version 4.0.

### Statistical analysis

Statistical analyses were performed with MedCalc version 12 (MedCalc software, Belgium). Differences between two groups were analyzed by Fisher’s exact test or by the chi-square test. Kaplan-Meier analysis was performed to estimate the distributions of PFS and OS in all patients. Cox proportional-hazard regression analysis was performed to calculate HR and 95% confidence interval (CI) for each clinicopathological factor. All differences were regarded as statistically significant when P < 0.05.

### Ethical statement

This study was approved by the Ethics Committee of Tohoku University Hospital.

## Results

### Patient characteristics

A total of 42 patients with advanced bone or soft tissue sarcoma received GD therapy in our hospital between October 2006 and September 2015. The clinicopathological characteristics of the patients are shown in Table 1. The median age was 54.5 years (range, 19 to 81 years). The patients included 23 men and 19 women. Among them, 2 had no prior chemotherapy, 29 had one, and 11 had two prior chemotherapies (mean, 1.2 regimens).

**Table 1:** Clinicopathological characteristics of 42 patients with advanced bone or soft tissue sarcoma.

Characteristics	N	%
Total	42	
Age		
Median	54.5	
Range	19–81	
Sex		
Men	23	54.8
Women	19	45.2
Primary origin		
Bone	8	19.0
Soft tissue	34	81.0
Primary site		
Extremity/trunk	23	54.8
Retroperitoneal/abdominal	15	35.7
Other^a^	4	9.5
Histology		
Malignant peripheral nerve sheath tumor	8	19.0
Leiomyosarcoma	7	16.7
Liposarcoma	5	11.9
Osteosarcoma	5	11.9
Synovial sarcoma	4	9.5
Desmoplastic small round cell tumor	3	7.1
Undifferentiated pleomorphic sarcoma	3	7.1
Chondrosarcoma	2	4.8
Ewing sarcoma	2	4.8
Alveolar soft part sarcoma	1	2.4
Chordoma	1	2.4
Fibrosarcoma	1	2.4
Prior lines of chemotherapy		
0	2	4.8
1	29	69.0
2	11	26.2
3	0	0.0
Mean	1.2	

^a^ Includes three intracranial and one unknown primary

Eight patients had bone sarcoma, and 34 had soft-tissue sarcoma. The primary tumor site was the extremities or trunk in 23 patients, retroperitoneal or abdominal in 15 patients, and other sites in 4 patients. Histological subtypes included malignant peripheral nerve sheath tumors (n = 8), leiomyosarcoma (n = 7), liposarcoma (n = 5), osteosarcoma (n = 5), synovial sarcoma (n = 4), desmoplastic small round cell tumor (n = 3), undifferentiated pleomorphic sarcoma (n = 3), chondrosarcoma (n = 2), Ewing sarcoma (n = 2), alveolar soft part sarcoma (n = 1), chordoma (n = 1), and fibrosarcoma (n = 1). Two patients had no prior chemotherapy, 29 had one regimen, and 11 had two. All patients who had one or two prior regimens had received doxorubicin or doxorubicin-based combination regimen. The mean number of prior regimens was 1.2.

### Efficacy

**Fig 1:**
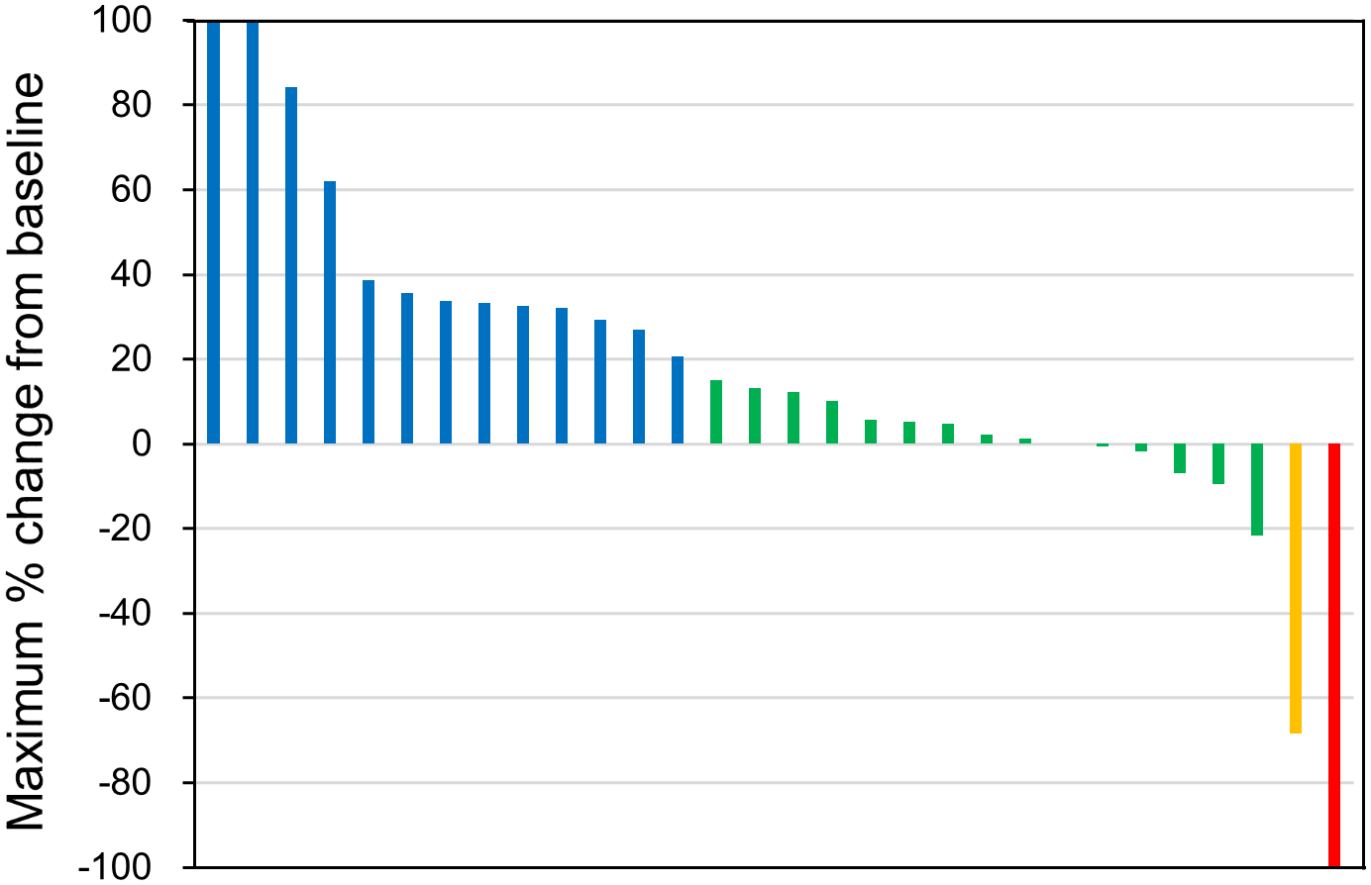
Waterfall plot of maximum percentage reduction in sizes of measurable lesions. Blue, green, yellow, and red columns represent progressive disease, stable disease, partial response, and complete response, respectively.

Among the 42 patients enrolled in this study, 29 patients had one or more measurable lesions. Among these 29 patients, CR was observed in one patient, PR in one, SD in 14, and PD in 13. RR was 6.9% and DCR was 55% (Table 2). Seven patients (24%) showed tumor reduction (Fig 1). In seven bone sarcoma patients with measurable lesions, CR was observed in one patient, SD in five, and PD in one. RR was 14% and DCR was 86%. In 22 soft tissue sarcoma patients with measurable lesions, PR was observed in one patient, SD in nine, and PD in 12. RR was 4.5% and DCR was 46%.

**Table 2:** Overall response of patients with measurable lesions.

	This study						Maki et al.^12)^	
	All		Bone sarcoma		Soft tissue sarcoma		Soft tissue sarcoma	
	N	%	N	%	N	%	N	%
Best response								
CR	1	3.4	1	14.3	0	0.0	2	2.9
PR	1	3.4	0	0.0	1	4.5	10	14.5
SD	14	48.3	5	71.4	9	40.9	39	56.5
PD	13	44.8	1	14.3	12	54.5	18	26.1
Total	29		7		22		69	
RR		6.9		14.3		4.5		17.4
DCR		55.2		85.7		45.5		71.0

CR, complete response; PR, partial response; SD, stable disease: PD, progressive disease, RR, response rate, DCR, disease control rate

In our study, DCR in bone sarcoma was quite high (86%; Tables 2 and 3). Among patients with soft tissue sarcoma, DCR was relatively high in those with malignant peripheral nerve sheath tumors (80%; Table 3), whereas there was no disease control in four patients with liposarcoma. In leiomyosarcoma, DCR was 33%, with one patient with SD and two patients with PD.

**Table 3:** Overall response according to histological subtypes.

	Bone			Soft tissue						
	OS	CS	CD	MPNST	LMS	LPS	SS	DSRCT	UPS	ASPS
CR	0	1	0	0	0	0	0	0	0	0
PR	0	0	0	0	0	0	0	0	1	0
SD	4	0	1	4	1	0	2	1	0	1
PD	1	0	0	1	2	4	1	2	2	0

OS, osteosarcoma; CS, chondrosarcoma; CD, chordoma; MPNST, malignant peripheral nerve sheath tumor; LMS, leiomyosarcoma; LPS, liposarcoma; SS, synovial sarcoma; DSRCT, desmoplastic small round cell tumor; UPS, undifferentiated pleomorphic sarcoma; ASPS, alveolar soft part tissue sarcoma.

**Fig 2:**
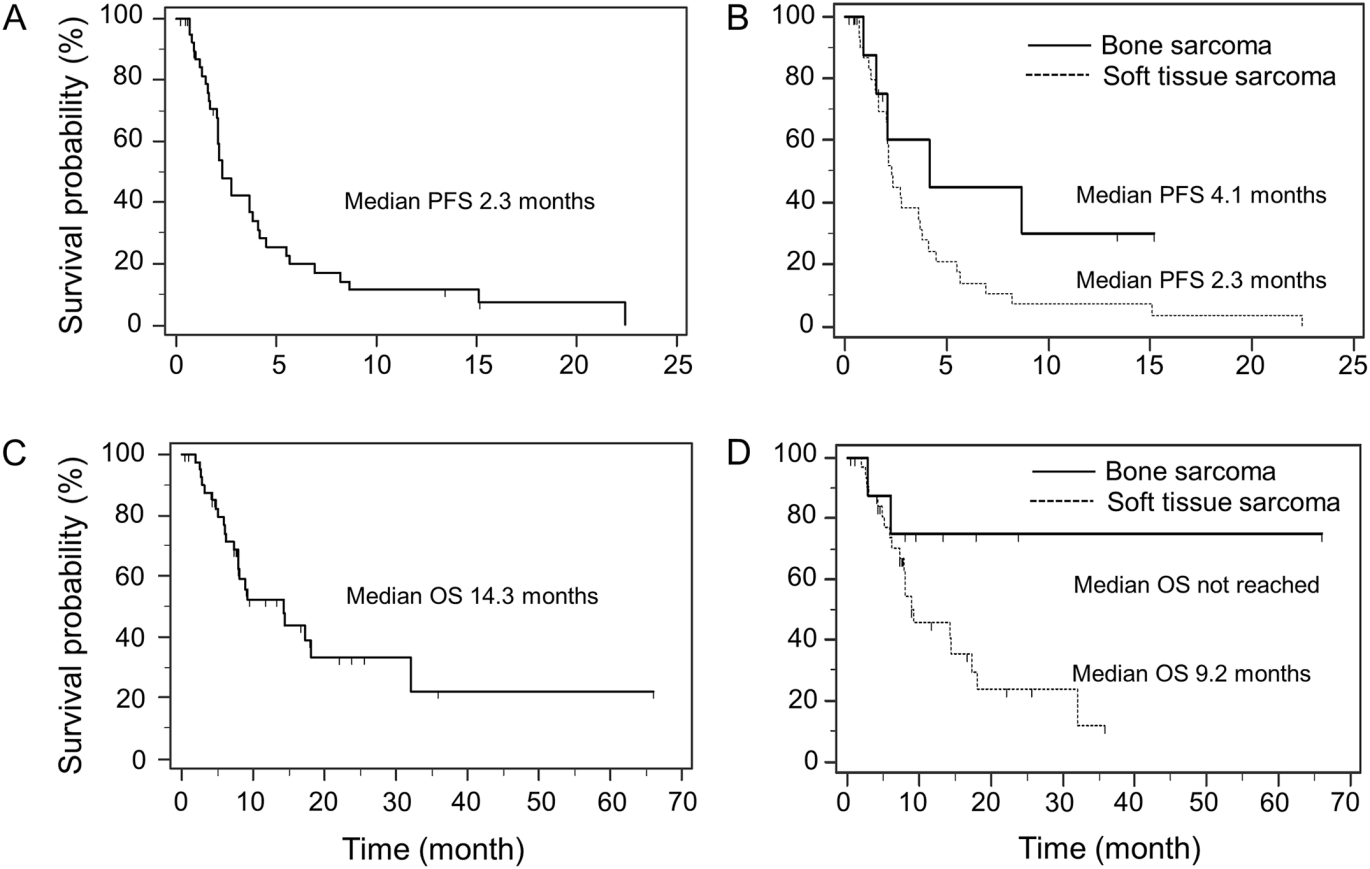
Progression-free survival and overall survival of the patients enrolled in this study. Progression-free survival (PFS) of all patients (A), and PFS between patients with bone sarcoma and patients with soft tissue sarcoma (B). Overall survival (OS) of all patients (C), and OS between patients with bone sarcoma and those with soft tissue sarcoma (D).

As shown in Fig 2, in all 42 patients, the median PFS and OS were 2.3 months (95% CI, 2.1 to 3.8) and 14.3 months (95% CI, 8.0 to 32.0), respectively. In patients with bone sarcoma, the median PFS was 4.2 months (95% CI, 1.6 to 8.7), and the median OS was not reached. In patients with soft tissue sarcoma, the median PFS and OS were 2.3 months (95% CI, 2.0 to 3.7) and 9.2 months (95% CI, 7.3 to 17.3), respectively. We next elucidated whether some clinicopathological factors had possible prognostic significance by using Cox proportional-hazard model. In univariate analyses for PFS, each factor such as sex (men vs. women, HR 1.22, 95%CI 0.61–2.44, P = 0.58), age (50 or older vs. 49 or younger, HR 1.23, 95%CI 0.61–2.47, P = 0.56), primary organ (bone vs. soft tissue, HR 0.46, 95%CI 0.18–1.21, P = 0.12), histological type (leiomyosarcoma vs. others, HR 1.34, 95%CI 0.54–3.32, P = 0.53), or the number of previous lines of chemotherapy (2 or more vs. 0–1, HR 1.67, 95%CI 0.70–3.96, P = 0.24) was not statistically significantly associated with PFS. Similarly, these factors including sex (HR 1.29, 95%CI 0.56–2.99, P = 0.56), age (HR 1.12, 95%CI 0.48–2.61, P = 0.79), primary organ (HR 0.30, 95%CI 0.07–1.27, P = 0.10), histological type (HR 1.53, 95%CI 0.56–4.20, P = 0.41), or the number of previous lines of chemotherapy (HR 1.41, 95%CI 0.50–3.93, P = 0.52) were not significantly associated with OS. Among these factors, only primary organ (bone vs. soft tissue) tended to associate with PFS or OS, although the difference was not statistically significant, probably due to the small number of patients, particularly with bone sarcoma, enrolled in this study.

Of note is a particular patient with dedifferentiated chondrosarcoma of the rib who obtained a CR (Table 3). Six months after the initial resection of primary tumors of the rib, the patient had developed four lung metastases in both lobes. After six courses of doxorubicin and ifosfamide, the four metastases were shrunk to some extent (SD) with no new lesions; they were then surgically resected. However, two months later, the patient had a second lung recurrence in the right S1. One year after the second resection of the lung lesion, she had third recurrence, with two metastases in the right upper lobe of the lung. She received three courses of GD, and obtained a good PR, determined by computed tomography. The pathological findings after the third resection of the lung metastases revealed an absence of viable malignant cells (pathological CR). The patient was then disease-free for 26 months, until developing lung metastases for the fourth time. A further four courses of GD led to a PR, and then the patient had the lung metastases resected. Finally, nine months after one more resection of a recurrent lesion of the chest wall, the patient had a recurrence of pleural dissemination of chondrosarcoma, but was still alive when the analysis closed. Since the first course of GD, the patient had survived for over 5.5 years.

A PR was obtained in a patient with undifferentiated pleomorphic sarcoma of the right thigh (Table 3). One year after the first resection of the primary tumors followed by adjuvant therapy with doxorubicin and ifosfamide, the patient had a recurrence in the right lower lobe of the lung. Three months after the metastasis was resected, the patient had a second recurrence in the right middle lobe of the lung. This was resected as well, and after six months, perioperative chemotherapy with doxorubicin and cisplatin, and two more resections of the recurrent lung metastases, the patient had a fifth recurrence of lung metastases. The patient then started receiving GD therapy; at the time of analysis, the efficacy had been sustained for 20 months with the best response of PR.

Among the patients who had SD, one patient with osteosarcoma of the mandible successfully had a good outcome. One and a half years after receiving a surgical resection of the osteosarcoma of the mandible, the patient had a recurrence in the right upper lobe of the lung. Eight months after resection of this metastasis and adjuvant chemotherapy composed of doxorubicin and cisplatin, the patient had two recurrent lung tumors in the right S6 and S9. The patient received a total of 32 courses of GD with the best response of SD during 15 months, and then had a resection of the metastases. Since then, the patient has been disease-free for over 15 months.

### Toxicity and tolerability

As shown in Table 4, grade 3 or more neutropenia was observed in 31 of 42 (74%) patients, and grade 3 or more thrombocytopenia occurred in 5 of 42 (12%) patients. Grade 4 neutropenia was observed in 12 of 42 (29%) patients, and grade 4 thrombocytopenia was seen in 2 of 42 (4.8%) patients. Febrile neutropenia occurred in 2 of 42 (4.8%) patients. Nine of 42 (21%) patients received G-CSF for therapeutic use against grade 4 neutropenia or grade 3 febrile neutropenia.

**Table 4:** Toxicities and dose reduction rate of the patients.

Factors	Grade	Incidence (%)
Hematological toxicity		
Neutropenia	≧ 3	74
	4	29
Thrombocytopenia	≧ 3	12
	4	4.8
Febrile Neutropenia		4.8
Non-hematological toxicity		
All	Any	33
Anorexia		17
Fatigue		12
Nausea		7.1
Rash		4.8
Stomatitis		4.8
All	≧ 3	2.4^a^

^a^anorexia in one patient

Non-hematological toxicities of any grade were observed in 14 of 42 (33%) patients. Anorexia was observed in 7 patients (17%), fatigue in 5 (12%), nausea in 3 (7.1%), rash in 2 (4.8%), and stomatitis in 2 (4.8%) (Table 4). In terms of severe toxicities of grade 3 or more, grade 3 anorexia was observed only in one patient (2.4%). No treatment-related death occurred.

Doses were reduced by at least one level (about 20% reduction of both gemcitabine and docetaxel) in 25 of 42 (60%) patients. Of the 41 patients who discontinued therapy, the reasons for discontinuation were PD in 34 (83%) patients, myelosuppression in 3 (7.3%), or other reasons in 4 (9.8%), respectively.

## Discussion

There is a growing need for more treatment options for patients with advanced bone and soft tissue sarcoma, a rare malignancy for which there are few effective regimens. Some new second- or later-line treatment options, including pazopanib, trabectedin, and eribulin, have recently emerged for soft tissue sarcoma [5, 15, 17]; however, the efficacies of these drugs are still limited and their efficacies in bone sarcoma are still unclear. The results of this retrospective analysis indicate that GD is an effective and tolerable second-line regimen for patients with advanced bone and soft tissue sarcoma in a practical daily setting.

For soft tissue sarcoma, the efficacies of GD in terms of RR, DCR, PFS, and OS were not good as those observed in a phase II study reported by Maki et al (RR 17%, DCR 71%, PFS 6.2 months, and OS 17.9 months)[12]. The reason for this may be that our study included fewer patients with leiomyosarcoma, particularly of the uterus (n = 1), or undifferentiated pleomorphic sarcoma (n = 3), which might have a higher sensitivity to GD as shown in previous studies [11, 12, 19, 20]. In a phase II study reported by Pautier et al [19], RR was 24% in uterine leiomyosarcoma, but only 5% in other leiomyosarcomas. In our study, RR was comparable to that of other leiomyosarcoma in Pautier’s study. Another reason may be the lower dose of docetaxel (70 mg/m^2^ in this study as compared with 100 mg/m^2^ plus prophylactic support of G-CSF [11, 12]). As 70 or 75 mg/m^2^ was the maximum approved dose in Japan, we used 70 mg/m^2^ of docetaxel in our cohort of patients. The possibility cannot be excluded that a dose of 100 mg/m^2^ may be more effective than that of 70 mg/m^2^. Although prospective trials are required to elucidate whether 70 mg/m^2^ of docetaxel is similarly effective to 100 mg/m^2^ of docetaxel with prophylactic support of G-CSF, considering our findings in this study that gemcitabine and 70 mg/m^2^ of docetaxel therapy showed some efficacy without more febrile neutropenia, we propose that gemcitabine and 70 mg/m^2^ of docetaxel combination therapy is enough feasible in Asian populations, and possibly in Caucasian populations.

In this study, myelosuppression was more frequently observed and resulting dose reduction was more frequently performed (60%) as compared with previous reports [11, 12], probably due to the absence with prophylactic support of G-CSF in this study. Non-hematological toxicities were less frequently observed, probably due in part to the lower initial dose and subsequent dose reduction of docetaxel. Treatment cessation due to toxicity was less frequent. As a result, the majority of the patients were able to continue GD therapy safely.

For bone sarcoma, although the number of patients with response evaluable lesions was low in this study (n = 7), RR (14%) and DCR (86%) seemed comparable to those previously reported by other groups (RRs of 9–29% and DCRs of 41–57%) [21–24, 26]. Our results and the results reported by other groups, that is, that GD therapy seems to be more effective in bone sarcoma than in soft tissue sarcoma, suggest that GD therapy should be a preferred option, at least in some populations of patients with bone sarcoma. However, since the number of patients with bone sarcoma was small in this study, the efficacy of GD therapy for patients with bone sarcoma should be further validated in future larger studies.

Among all patients, RR was relatively low (6.9%), but it should be noted that some responders experienced long survival. One patient with dedifferentiated chondrosarcoma had a CR followed by surgical resection for lung metastasis and survived for over 5 years. A patient with undifferentiated pleomorphic sarcoma maintained a PR for 20 months. Another patient with osteosarcoma has been disease-free for over 15 months, since resection of lung metastases after 32 maintenance courses of GD. These favorable outcomes in some responders, together with results from other reports [18, 19], prompt us to believe that GD therapy should remain as one of the treatment options at least in a subpopulation of patients with bone or soft tissue sarcoma who benefit from this therapy. In addition, more recently, Tanaka et al. have shown the feasibility and efficacy of GD in Japanese patients with 9.7% of RR and 4.8 months of median PFS, and our data in the present study generally supports their results [27]. Moreover, a recent phase II trial has shown that GD with the addition of bevacizumab resulted in a good RR (49%) in 35 patients with leiomyosarcoma, undifferentiated pleomorphic sarcoma, angiosarcoma, and pleomorphic liposarcoma, suggesting that some molecularly-targeted drugs may increase the efficacy of GD [28].

Our study has several limitations. First, it was a retrospective, non-randomized study. In particular, data about toxicities was limited, since the data was retrospectively obtained from medical records. Second, the number of patients enrolled was relatively small. Nonetheless, it should be noted that in this study, a considerable proportion of patients achieved SD and some patients even achieved a CR or PR after doxorubicin-based therapy with manageable toxicities and long survival, in daily practical settings.

In conclusion, our data support the notion that GD therapy is an appropriate option for salvage-line therapy for patients with advanced bone and soft tissue sarcoma, including Asian populations. Future larger and prospective trials are warranted to establish the efficacy and safety of GD therapy in bone and soft tissue sarcoma.

## Supporting information

S1 Table Clinicopathological data of 42 patients with bone or soft tissue sarcoma. (XLSX)
